# The value of prognostic factors for uterine cervical cancer patients treated with irradiation alone

**DOI:** 10.1186/1471-2407-7-234

**Published:** 2007-12-22

**Authors:** Rūta Grigienė, Konstantinas P Valuckas, Eduardas Aleknavičius, Juozas Kurtinaitis, Simona R Letautienė

**Affiliations:** 1 Department of Diagnostic Radiology, Oncology Institute of Vilnius University, Santariskiu 1, Vilnius, Lithuania; 2 Clinic of Conservative Tumour Therapy, Oncology Institute of Vilnius University, Santariskiu 1, Vilnius, Lithuania; 3 Cancer Control and Prevention Center, Oncology Institute of Vilnius University, Santariskiu 1, Vilnius, Lithuania

## Abstract

**Background:**

The aim of our study was to investigate and evaluate the prognostic value of and correlations between preclinical and clinical factors such as the stage of the disease, blood Hb level before treatment, size of cervix and lymph nodes evaluated by CT, age, dose of irradiation and duration of radiotherapy related to overall survival, disease-free survival, local control and metastases-free survival in cervical cancer patients receiving radiotherapy alone.

**Methods:**

162 patients with International Federation of Gynecology and Obstetrics (FIGO) stage IIA-IIIB cervical carcinoma treated with irradiation were analysed. Univariate and multivariate analyses using the Cox regression model were performed to determine statistical significance of some tumor-related factors.

**Results:**

The Hb level before treatment showed significant influence on overall survival (p = 0.001), desease free survival (p = 0.040) and local control (p = 0.038). The lymph node status (>10 mm) assessed on CT had impact on overall survival (p = 0,030) and local control (p = 0,036). The dose at point A had impact on disease free survival (p = 0,028) and local control (p = 0,021) and the radiotherapy duration had showed significant influence on overall survival (p = 0,045), disease free survival (p = 0,006) and local control (p = 0,033).

**Conclusion:**

Anemia is a significant and independent prognostic factor of overall survival, disease-free survival and local control in cervical cancer patients treated with irradiation. The size of lymph nodes in CT is an independent prognostic factor for overall survival and local control in cervical cancer patients. The size of cervix uteri evaluated by CT has no prognostic significance in cervical cancer patients treated with radiotherapy. The prognostic value of FIGO stage of cervical cancer is influenced by other factors, analyzed in this study and is not an independent prognostic factor.

## Background

In 2004, 569 new cases of cervical cancer were diagnosed in Lithuania. This number is higher (by about 100 cases) than that observed in 2003. The lifetime risk of cervical cancer in Lithuania is 1 of 65 women. Most often cervical cancer develops in women aged 30 to 59 years, i.e. when they are most able-bodied. In 33% of patients stage I and in 21% – stage II were diagnosed. Advanced cervical cancer (stages III and IV) was diagnosed in 46% of patients [1]. Radiotherapy was usually used in stages II-IV cervical cancer. About 60% of patients with cervical cancer in Lithuania used to undergo radiotherapy. Now the golden standard in advanced cervical cancer patients treatment is chemoradiotherapy, but this treatment option is more toxic and may be not everyone needs such aggressive treatment. Therefore it is very important to define the factors that influence results in radiotherapy.

The FIGO (The International Federation of Gynecology and Obstetrics) classification [2] is used to define the stage of the cervical cancer. Once established, the stage should not be changed. Clinical examination of the patient is of crucial importance in the staging of cervical cancer. Stage is determined according to tumor extension beyond the uterine cervix and its invasion into adjacent tissues: parametric, pelvic wall, vagina, bladder and rectum. Radiological investigations involve only chest X-ray for evaluating lung metastases and intravenous urograms for determining the presence of hydronephrosis. No modern methods of radiological investigations such as CT, MRI, PET are used for the staging of cervical cancer according to FIGO classification [3-8].

According to the current FIGO classification, cancer patients are divided into groups, and 5 – year survival within these groups is different [9,10]. The incidence levels differ significantly within every stage [11-14]. Therefore, it could be assumed that the present criteria of staging are insufficient, and there are some other factors that influence survival; these factors should be taken into consideration when selecting treatment options [15-18]. The goal of our study was to determine the factors influencing the survival of advanced cervical cancer patients.

## Methods

This was a cohort retrospective study. Case histories of 350 patients treated with radiotherapy due to stage II and III cervical cancer at the Institute of Oncology, Vilnius University in 1999–2003 were analyzed. 162 patients who met inclusion and exclusion criteria were involved into the study.

Inclusion criteria:

• Histologically confirmed cervical cancer

• FIGO stage II-III

• Only combined radiotherapy (EBRT+Brachytherapy)

• Pelvic CT scan before treatment

• The treatment was performed using a linear accelerator.

Exclusion criteria:

• Follow-up duration <1 year

• Treatment started before 1999

• Second primary tumor (except skin non-melanomas).

The censoring time was March 2005. The minimal duration of follow-up was one year (365 days), the maximal being 5.8 years (2108 days), mediana – 2.7 years (992 days (95% CI 911–1127), 32 months. The patients' age ranged from 21 to 81 years. The distribution by age groups was the following: younger than 50 years – 74 patients (45.7%), from 50 to 64 – 60 (37%), older than 64 years – 28 (17,3%). The median age was 50 years (95% CI, 49.0–54.0), average age being 52 years. The stage was evaluated by the oncologist-gynecologist according to FIGO criteria. There were 69 patients with stage II (43.6%) and 93 patients with stage III (57.4%). According to the morphology of tumor the patients were distributed into 5 groups: squamous cell carcinoma G1 – 4 (2.2%), squamous cell carcinoma G2 – 37 (20.4%), squamous cell carcinoma G3 – 137 (75.1%), adenocarcinoma – 2 (1.1%); other – 1 (0.5%). Blood count was performed and blood Hb level evaluated in all patients before treatment. According to the results of this test the patients were divided into two groups: Hb <120 g/l and Hb > 120 g/l. There were 68 patients with a lower level of Hb (<120 g/l) (42.0%), and 94 patients with Hb>120 g/l (58.0%).

Combined radiotherapy involving external irradiation and intracavitary radiotherapy was applied to all patients. They were irradiated with 25 MeV photons using a linear accelerator and four adapted fields with a blocking system ("box" method). The upper border of the field was at the level of L4-L5 intervertebral space, the lower border being at the level of the middle of the obturator foramen. The lateral borders of frontal fields involve 1 cm of the bone pelvis. Single focal dose (SFD) of external irradiation was 2 Gy. Patients were irradiated 5 days a week. The doses were calculated for points A and B. Point A is located 2 cm aside from the middle line and 2 cm above the lateral vaults of the vagina. This is the point of cervix uteri that corresponds to the top of the paracervical triangle. Point B is located 5 cm aside from the middle line and 2 cm above the lateral vaults of the vagina. This is the point in the parametrium. For the majority of patients the dose of EBRT (2 Gy per fraction) was prescribed to the central axis point of beam intersection. Box technique was employed for these patients. Dose homogenity throughout the CT outlined treatment volume satisfied ICRU 50 requirements. For some patients, however, the central blocking from AP-PA fields was used. The prescription point (2 Gy per fraction) in this case was located 1 cm laterally from the physical edge of the block. The lowest dose of external irradiation at point A was 23 Gy, the highest dose at this point being 55 Gy; the median dose was 37 Gy (95% CI, 35.6 – 38.4). The lowest dose of external irradiation at point B was 28 Gy, the highest dose being 60 Gy; the median dose was 50 Gy (95% CI 49.1–50.9). For intracavitary irradiation, an AGAT-VU irradiation machine with a Co60 source (1,25 MeV energy) and the Fletcher type applicator was used. The average fraction dose was 7 Gy. One to six fractions (on average 4 fractions) were applied. The procedure was performed once a week. The lowest dose of intracavitary irradiation at point A was 8 Gy, and the highest dose at this point was 48 Gy, the median dose was 40 Gy (95% CI, 38.1 – 41.9). The lowest dose at point B was 2 Gy, the highest dose being 20 Gy and the median dose was 10 Gy (95% CI, 8.8 – 11.2). Total treatment dose (EBRT+Brachytherapy) for patients described in the study was 76 Gy (median) for the point A and 60 Gy (median) for the point B.

In the Institute of Oncology, Vilnius University, non-contrast computed tomography of pelvic organs is performed during pre-treatment planning of cervical cancer patients undergoing radiotherapy. We used a General Electric Syntec Synergy computed tomography machine without spiral software. CT scans were performed from the upper edge of the ileum wing down to the ischial tuberosity; the axial slices were made with slice spaces of 10 mm. The status of pelvic organs and the size of pelvic lymph nodes were assessed. The smallest size of all visible lymph nodes was measured. The patients were assigned to one of the three study groups according to the results of these measurements: 73 patients without CT evidence of pelvic lymph nodes (45.0%), 51 patient with lymph nodes from 1 to 10 mm (31.5%); and 38 patients with lymph nodes 10 mm and larger. The size of cervix, its greatest diameter was also measured. By this factor the patients were divided into two study groups: size of cervix < 60 mm, and size of cervix ≥60 mm. There were 81 patients (50.0%) in the first and 71 (4.8%) in the second group. In 10 patients cervix size has been not recorded and there were no respective data in patients' case histories. The size of cervix but not the size of tumor was selected, because the resolution of soft tissues in computed tomography is not sufficient to evaluate a single tumor precisely. Since this method is used in our everyday work for pre-treatment planning, our goal was to clarify how this method alone could be used for the prognosis of the course of the disease.

We analyzed how such factors as the stage of the disease, blood Hb level before treatment, size of cervix and lymph nodes evaluated by CT, age, dose of irradiation and duration of radiotherapy influence the overall survival, disease-free survival, local control and distant-metastases-free survival. Overall survival was calculated evaluating the time from the end of treatment to the last follow-up or death. Disease-free survival was evaluated calculating the time from the end of treatment to any disease progression (local recurrence or distant metastases). Local control was evaluated calculating the time from the end of treatment to any recurrence in the pelvis. Distant-metastases-free survival was evaluated calculating the time from the end of treatment to any disease progression outside the pelvis. Survival analysis was performed using the Kaplan-Meier method. Differences in the rate and their significance were evaluated using the log-rank test (p < 0,05). Interrelations of the analyzed factors were evaluated using cross-tabulation and the Cox proportional hazard model. Probability of complications after radiotherapy was assessed using the probit method. Statistical analysis was performed using the STATA software.

### Ethics

The study protocol was reviewed and approved by the Lithuanian Bioethical Committee and complied with the recommendations of the Declaration of Helsinki for biomedical research involving human subjects.

## Results

The Kaplan-Meier method was used to assess the importance of factors we have selected (age, stage of the disease, Hb blood level before treatment, size of cervix and lymph nodes evaluated on CT, irradiation dose and radiotherapy duration) influencing the overall survival, disease-free survival, local control and distant-metastases-free survival of patients with cervical cancer (Table 1).

**Table 1:** Univariate analysis of prognostic factors by Cox proportional hazard model: overall survival and disease-free survival at 3 years

Factor	Overall survival RR (95% CI)	p value	Disease-free survival RR (95% CI)	p value
Age	0.98 (95% CI, 0.96–1.01)	0.2735	0.98 (95% CI, 0.96–1.01)	0.231
Stage	3.96 (95% CI, 1.90–8.24	0.0000	2.81 (95% CI, 1.40–5.63)	0.004
L/node size by CT	1.62 (95% CI, 1.14–2.32)	0.0078	1.54 (95% CI, 1.06–2.23)	0.023
Cervix size by CT	1.41 (95% CI, 0.85–2.33)	0.1754	1.03 (95% CI, 0.72–2.01)	0.478
Hb blood level	0.27 (95% CI, 0.15–0.51)	0.000	0.39 (95% CI, 0.21–0.73)	0.003
Dose at point A	0.90 (95% CI, 0.50–1.62)	0.7360	0.98 (95% CI, 0.53–1.81)	0.953
Dose at point B	1.49 (95% CI, 0.78–2.83)	0.2144	1.74 (95% CI, 0.87–3.46)	0.117
Duration of treatment	1.85 (95% CI, 1.24–2.76)	0.0024	2.06 (95% CI, 1.34–3.15)	0.001

The overall survival for the whole cohort of our study was 71,7% (95%CI 63.7%–78.3%) and disease-free survival was 74,2% (95%CI 66.2%–80.7%).

**Figure 1:**
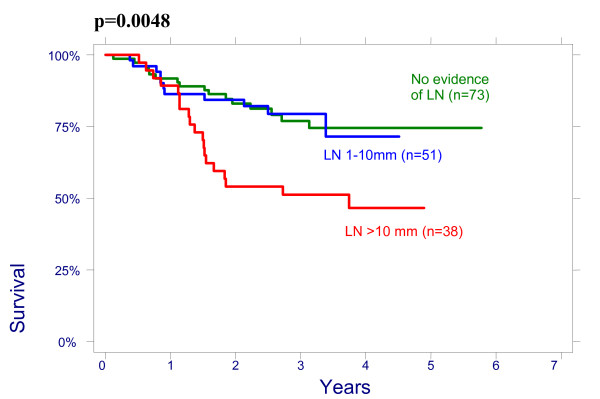
Overall survival according to lymph nodes (LN) size on CT.

**Figure 2:**
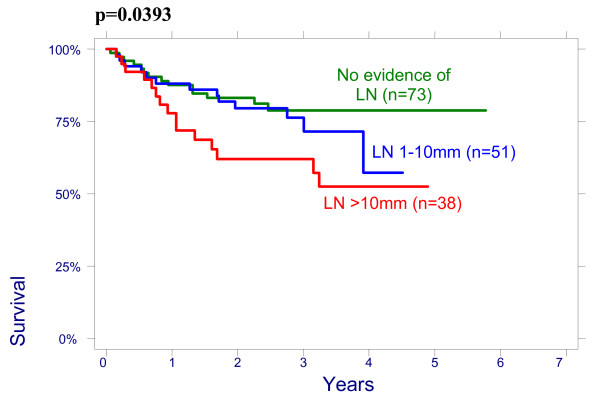
Disease free survival according to lymph nodes (LN) size on CT.

**Figure 3:**
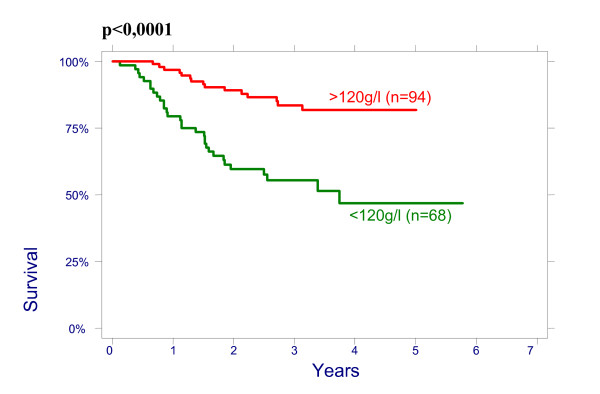
Overall survival as a function of initial hemoglobin level.

**Figure 4:**
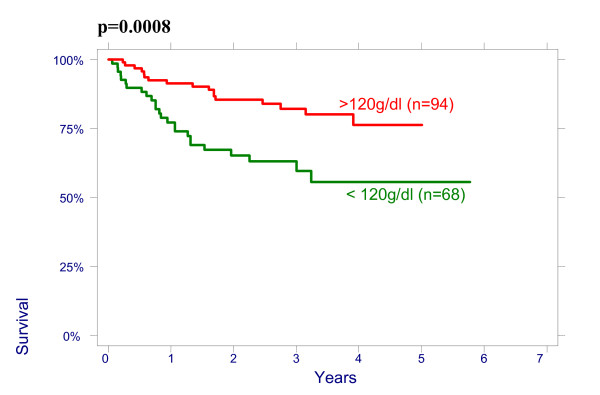
Disease-free survival as a function of initial hemoglobin level.

**Figure 5:**
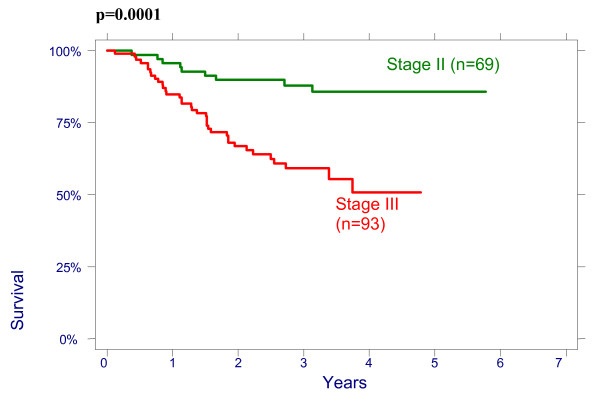
Overall survival according to FIGO stage.

**Figure 6:**
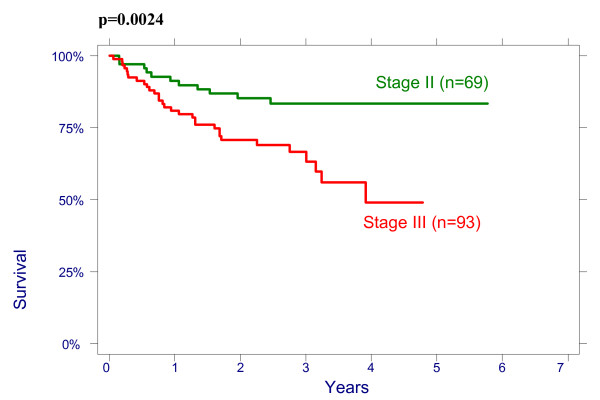
Disease-free survival according FIGO stage.

**Figure 7:**
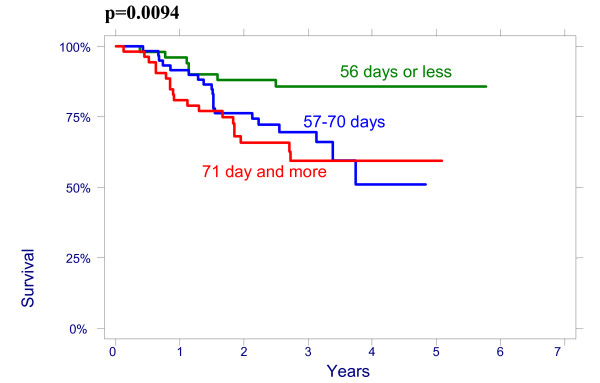
Overall survival as a function of radiation treatment duration.

**Figure 8:**
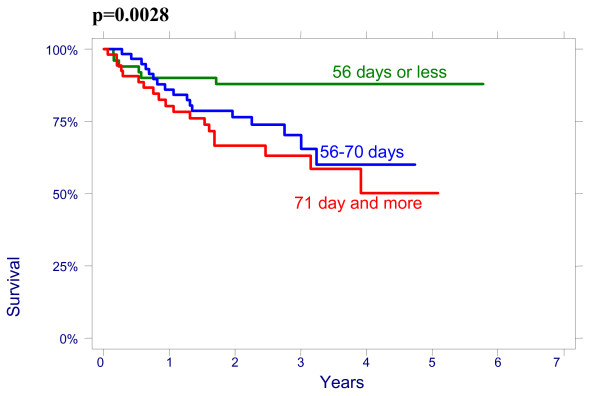
Disease-free survival as a function of radiation treatment duration.

There were no significant effect of patient age on overall survival (OS): patients < 50 had a 3-year OS of 64% (95%CI 51.3%–74.3%), 78,8% (95%CI 65.6%–87.4%) for age 50–64 and 76.3% (95%CI 54.2%–88.8%) for > 64 years (p = 0.2960); and on disease free survival (DFS): patients < 50 had a 3-year DFS of 70.7% (95%CI 58.1%–80.13%), 76.5% (95%CI 62.8%–85.8%) for age 50–64 and 79.8% (95%CI 57.4%–91.2%) for > 64 years (p = 0.4513). There were also no significant differences between the patients groups with different size of the cervix evaluated by CT on OS and on DFS: patients <60 mm had a 3-year OVS of 75.3% (95%CI 63.9%–83.5%), DFS of 74.4% (95%CI 62.9%–82.8%) and patients ≥60 mm had a 3-year OS 66.4% (95%CI 53.2%–76.7%), DFS 69.2% (95%CI 56.9%–78.6%), p = 0.4882. The effect of lymph nodes (LN) size assessed by CT on OS is shown in Figure 1. Patients with no evidence of lymph nodes on CT had a 3-year OS of 76.9% (95%CI 64.2%–85.6%), 79.5% (95%CI 65.0%–88.5%) with lymph nodes being 1–10 mm and 51.2% (95%CI 34.2%–65.9%) with lymph nodes being ≥10 mm in his shortest axis on CT (p = 0.0048). We looked for lymph nodes size assessed by CT effect on DFS (Figure 2). Patients with no evidence of lymph nodes on CT had a 3-year DFS of 78.8% (95%CI 66.4%–87.0%), 76.2% (95%CI 60.7%–86.3%) with lymph nodes being 1–10 mm and 62.0% (95%CI 43.4%–76.0%) with lymph nodes being ≥10 mm (p = 0.0498). Initial hemoglobin levels <120 g/l were associated with a 55.4% (95%CI 42.2%–66.7%) OS and 71.0% (95%CI 57.8%–80.8%) DFS compared with 83.5% (95%CI 73.6%–90.9%) OS and 87.9% (95%CI 78.3%–93.4%) DFS if the hemoglobin was ≥120 g/l (Figures 3 and 4). OS and DFS as a function of FIGO stage were 87.9% (95%CI 77.1%–93.8%) and 83.3% (95%CI 71.8%–90.5%) for stage II, 59.1% (95%CI 47.6%–68.9%) and 66.6% (95%CI 54.6%–76.1%) for stage III (Figures 5 and 6). Radiation doses to point A of <76 Gy were associated with a 71.2% (95%CI 58.3%–80.8%) OVS and 71.7% (95%CI 57.7%–81.7%) DFS whereas with doses ≥76 Gy the OS was 72.6% (95%CI 61.8%–80.8%) and DFS was 75.9% (95%CI 57.8%–81.7%). Radiation doses to point B of <60 Gy were associated with 78.7% (95%CI 66.1%–87.0%) OS and 81.3% (95%CI 68.7%–89.2%) DFS whereas with doses ≥60 Gy the OS was 68.5% (95%CI 58.0%–76.9%) and DFS was 70.4% (95%CI 59.6%–78.8%). These differences were not statistically significant. Treatment durations of 56 days or less were associated with OS of 84.7% (95%CI 70.5%–92.4%) and with DFS of 86.9 (95%CI 73.0%–93.9%), 57–70 days were associated with OS of 72.6% (95%CI 60.1%–81.7%) and with DFS of 73.3% (95%CI 60.0%–82.9%) compared with OS of 53.8% (95%CI 35.7%–68.8%) and DFS of 58.2% (95%CI 39.7%–72.9%) in patients with duration of more than 71 day (Figures 7 and 8).

We used the Cox proportional hazard model to evaluate the significance of the factors we have selected (age, stage of the disease, blood Hb level before treatment, size of cervix and lymph nodes evaluated by CT, irradiation dose and radiotherapy duration) influencing the overall survival, disease-free survival, local control and distant-metastases-free survival of cervical cancer patients (univariate analysis, Table 1 and 2); we have also used this method to assess how the prognostic significance of these factors depends on the other factors analyzed (multivariate analysis, Table 3 and 4).

**Table 2:** Univariate analysis of prognostic factors by Cox proportional hazard model: local control and distant metastases-free survival

Factor	Local control RR (95% CI)	p value	Distant-metastases-free survival RR (95% CI)	p value
Age	0,98 (95% CI, 0.95–1.01)	0.150	1.00 (95% CI, 0.96–1.03)	0.874
Stage	2.58 (95% CI, 1.14–5.84)	0.023	1.97 (95% CI, 0.78–5.03)	0.154
L/node size by CT	1.64 (95% CI, 1.05–2.56)	0.028	1.87 (95% CI, 1.08–3.21)	0.024
Cervix size by CT	1.29 (95% CI, 0.70–2.40)	0.420	1.05 (95% CI, 0.51–2.17)	0.895
Hb blood level	0.29 (95% CI, 0.14–0.63)	0.002	0.70 (95% CI, 0.29–1.69)	0.427
Dose at point A	0.81 (95% CI, 0.70–1.66)	0.569	1.82 (95% CI, 0.70–4.75)	0.222
Dose at point B	1.64 (95% CI, 0.73–3.67)	0.234	2.56 (95% CI, 0.85-7-69)	0.094
Duration of treatment	1.89 (95% CI, 1.14–3.13)	0.014	2.09 (95% CI, 1.14–3.83)	0.018

**Table 3:** Multivariate analysis of prognostic factors by Cox proportional hazard model: overall survival and disease-free survival at 3 years

Factor	Overall survival	P	Disease-free survival	P
Age	0.98 (95% CI, 0.97–1.02)	0.565	0.98 (95% CI, 0.95–1.01)	0.201
Stage	2.31 (95% CI, 0.98–5.40)	0.054	1.37 (95% CI, 0.62–3.04)	0.436
L/node size by CT	1.54 (95% CI, 1.04–2.28)	0.030	0.91 (95% CI, 0.51–160)	0.067
Cervix size by CT	1.06 (95% CI, 0.61–1.85)	0.834	0.50 (95% CI, 0.26–0.97)	0.732
Hb blood level	0.35 (95% CI, 0.18–0.67)	0.001	0.41 (95% CI, 0.18–0.91)	0.040
Dose at point A	0.48 (95% CI, 0.22–1.06)	0.070	1.67 (95% CI, 0.67–4.14)	0.028
Dose at point B	1.16 (95% CI, 0.47–2.88)	0.743	1.74 (95% CI, 0.87–3.46)	0.272
Duration of treatment	1.62 (95% CI, 1.01–2.61)	0.045	2.04 (95% CI, 1.23–3.40)	0.006

**Table 4:** Multivariate analysis of prognostic factors by Cox proportional hazard model: local control and distant metastases-free survival

Factor	Local control	P	Distant-metastases-free survival	p
Age	0,98 (95% CI, 0.94–1.01)	0.234	0.99 (95% CI, 0.95–1.03)	0.726
Stage	1.20 (95% CI, 0.47–3.07)	0.770	0.90 (95% CI, 0.30–2.68)	0.856
L/node size by CT	1.67 (95% CI, 1.03–2.70)	0.038	1.79 (95% CI, 0.97–3.31)	0.063
Cervix size by CT	0.87 (95% CI, 0.44–1.72)	0.693	0.82 (95% CI, 0.34–1.98)	0.656
Hb blood level	0.36 (95% CI, 0.16–0.80)	0.013	0.75 (95% CI, 0.28–1.97)	0.555
Dose at point A	0.34 (95% CI, 0.14–0.85)	0.021	0.73 (95% CI, 0.19–2.74)	0.642
Dose at point B	1.71 (95% CI, 0.60–4.88)	0.314	1.68 (95% CI, 0.37–7.51)	0.500
Duration of treatment	1.89 (95% CI, 1.05–3.40)	0.033	1.95 (95% CI, 0.90–4.23)	0.090

Univariate Cox regression analysis has shown (Table 1 and 2) that the stage of the disease, the lymph nodes size evaluated on CT, blood Hb level before treatment and radiotherapy duration are statistically significant prognostic factors for overall survival. Stage, the lymph nodes size evaluated on CT, blood Hb level before treatment and radiotherapy duration are statistically significant prognostic factors for disease-free survival. These factors are also statistically significant prognostic factors for local control survival. The size of lymph nodes evaluated by CT and the duration of radiotherapy are statistically significant prognostic factors for distant-metastases-free survival.

The multivariate Cox analysis has shown (Table 3 and 4) that the size of lymph nodes assessed by CT, blood Hb level before treatment and radiotherapy duration are independent prognostic factors for overall survival. Radiotherapy duration and blood Hb level are independent prognostic factors for disease-free survival. Although stage and the lymph nodes size evaluated by CT are statistically significant prognostic factors for disease-free survival when assessed alone, they lose their significance when analyzed together with other factors. The dose at point A has no prognostic significance when analyzed alone; however, it becomes statistically significant when analyzed together with other factors. Our data show that the size of lymph nodes evaluated by CT, blood Hb level before treatment and radiotherapy duration remain independent prognostic factors influencing local control. However, the dose at point A is of no prognostic significance for local control when analyzed alone, but it acquires a statistically significant prognostic value when analyzed together with other factors. None of the factors analyzed is an independent prognostic factor for distant-metastases-free survival. As the value of the CT size of lymph nodes remains almost unchanged, we assume that this factor is most precise for defining the probability of distant metastases.

## Discussion

Several prognostic factors influencing survival in cervical cancer patients have been established. Some of these factors are related to patients' characteristics (age, blood hemoglobin level), the others being related to tumor (stage, lymph node involvement, size of tumor) or treatment characteristics (irradiation doses, duration treatment) [19].

Analysis of various factors influencing survival in cervical cancer patients it usually presumed that cancer in younger patients is biologically much more aggressive than in older ones [20,21]. However, data of other authors, mostly those who analyzed surgical treatment of cervical cancer, showed that age is not the factor worsening the prognosis of survival [22]. These differences could occur due to selecting different treatment options. Surgical treatment is applied mostly to young women with small tumors. Women of the same age but with big tumors usually receive radiotherapy. Therefore, the poorer prognosis in this patients' group could be related not to age, but to other prognostic factors, e.g. size of the tumor. Our data confirm this presumption. In our study, age had no impact on the survival of cervical cancer patients treated with irradiation.

One of the most significant prognostic factors in cervical cancer is anemia. We have found that when analyzed together with other factors, blood Hb level is an independent prognostic factor for the overall survival, disease-free survival and local control; however, it has no prognostic value for distant-metastases-free survival. The mechanism of relation between anemia and poorer prognosis in cervical cancer patients is unclear. One of the hypotheses claims that in patients with poor prognosis anemia is present at the moment of diagnosis. Advocates of this theory affirm that tumor-related anemia is one of the signs of tumor aggressiveness, similar to weight loss and poor performance status [23]. In these cases, correction of blood Hb level during treatment will have no impact on the effect of treatment. Another explanation of the relationship between blood Hb level and prognosis of the disease could be poor tumor sensitivity to radiotherapy due to decreased oxygen supply. This hypothesis allows concluding that blood Hb level correction during treatment should improve treatment results [20].

There are data showing that the size of cervix evaluated in CT scans is directly related to the overall survival, disease-free survival, local control and distant-metastases-free survival [3,7]. Data of our study have shown, that the CT size of cervix has no prognostic significance for cervical cancer patients if only radiotherapy is applied. This discrepancy should be explained by a relatively low resolving capacity of CT as regards soft tissue. Therefore it is difficult to distinguish between specific tumorous infiltration of the parametrium and inflammation and to separate tumor from normal tissue. The goal of our study was to elucidate the factors influencing the clinical course without employing any additional clinical investigations except those routinely used in everyday practice. Therefore in our study CT was used for radiotherapy planning without contrast enhancement, as it defined by the treatment protocol. For this reasons the resolving capacity and accuracy of some data could decrease and errors could be bigger.

We have found that enlarged (more than 10 mm) lymph nodes found by CT before treatment are indicative of a shorter survival and of a more rapid disease progression of the cervical cancer patients. It has been proven that the prognostic value of the size of lymph nodes in CT is of the same significance for the progression of advanced cervical cancer as it is in early stages of the disease, when surgery is applied and the removed lymph nodes are morphologically evaluated [22]. Therefore we think that when selecting the treatment option for advanced cervical cancer these factors should be taken into account.

According to the literature, clinical stage is one of the main prognostic factors in cervical cancer patients [24]. Results of our study clearly show that the stage is not an independent factor which could help predict the clinical course; it also depends on the other factors which we have analyzed. Therefore we suppose that other factors such as size of lymph nodes evaluated by CT should also be taken into consideration when selecting the treatment option.

According to the literature, one of the major prognostic factors for cervical cancer treated by combined radiotherapy is irradiation dose at point A, which is applied directly to the tumor [15,24]. Analysis of the impact of irradiation dose at point A on survival has shown, that the higher irradiation dose is applied at point A, the smaller the probability of the disease progression, especially of local recurrence. Univariate and multivariate Cox's analysis has shown that irradiation dose at point B (tissues surrounding cervix uteri) has no prognostic significance. However, our study is a retrospective one; therefore it was difficult to evaluate all characteristics of radiotherapy. In this case, selection of radiotherapy doses was affected by a personal opinion of the managing physician, by the size of tumor and other possible factors.

At present, there are a lot of discussions how to reduce radiotherapy treatment time. According to the literature, the longer the duration of radiotherapy in cervical cancer, the shorter is the overall survival, disease-free survival, and local control [24,25]. Results of our study confirm the published data that radiotherapy duration is an independent prognostic factor for overall survival, disease-free survival, and local control. However, the impact of radiotherapy on the occurrence of distant metastases is related to the other prognostic factors we have analyzed. This effect of the duration of radiotherapy is related to accelerated clonogenic repopulation, which happens during radiotherapy and is especially active in cases of locally advanced cervical cancers [25]. We have found that the longer was the duration of treatment, the higher were irradiation doses at points A and B. It could be supposed that increasing the dose was an attempt to compensate for a gap in radiotherapy. However, our results clearly show that this is not a sufficient compensation.

At present, chemoradiotherapy is indicated to all patients with locally advanced (stage IIB-IVA) cervical cancer, regardless of other factors influencing the survival, which are taken into account in surgical treatment of early stages. Data of randomized trials involving 4580 patients showed that the most beneficial effect of chemoradiotherapy on survival in comparison with radiotherapy alone was observed in patients with metastases in pelvic lymph nodes and large tumor. Stages I and II were found in 68% of patients involved in clinical trials; most of them had stage IB tumor with metastases in pelvic lymph nodes and large tumors found during operations [19]. Data of clinical trials of patients with predominantly advanced cervical cancer showed that there was no significant survival differences in groups of chemoradiotherapy and radiotherapy alone [17,18]. In our study we analyzed prognostic factors for uterine cervical cancer treated with radiotherapy alone and we found some factors significantly influencing the results of treatment. Data of our study show that the size of lymph nodes in computed tomography is an independent prognostic factor in cervical cancer patients undergoing radiotherapy. Although there are no possibilities to confirm lymph node involvement as can be done during surgery, it could be assumed that the prognostic significance of this factor in advanced cancer is as high as in early stages. Therefore we suppose that nodal status must be taken into account while selecting the treatment strategy for patients with advanced cervical cancer and patient without lymph nodes involvement on CT don't need chemoradiotherapy. We propose including CT-evaluated lymph nodes as additional criteria during patients' assignment to different arms in clinical trials. This should be confirmed on prospective randomized trials.

## Conclusion

In conclusion, we can say that anemia is a significant and independent prognostic factor of overall survival, disease-free survival and local control in cervical cancer patients treated with irradiation. The size of lymph nodes in CT is an independent prognostic factor for overall survival and local control in cervical cancer patients. The size of cervix uteri evaluated by CT has no prognostic significance in cervical cancer patients treated with radiotherapy. The prognostic value of FIGO stage of cervical cancer is influenced by other factors, analyzed in this study and is not an independent prognostic factor.

## Competing interests

The author(s) declare that they have no competing interests.

## Authors' contributions

RG carried out the collection of the data, the statistical analysis with interpretation and drafted the manuscript. JK performed the statistical analysis and approved the final manuscript. KPV designed the concept of this study, performed radiation therapy for patients and participated in treatment coordination. EA performed radiation therapy for patients and participated in treatment coordination, revised the manuscript. SRL carried out the collection of the data, performed CT for patients, revised the manuscript. All authors were involved in the research presented and approved the final manuscript.

## Pre-publication history

The pre-publication history for this paper can be accessed here:


